# Death of Neurons following Injury Requires Conductive Neuronal Gap Junction Channels but Not a Specific Connexin

**DOI:** 10.1371/journal.pone.0125395

**Published:** 2015-05-27

**Authors:** Joseph D. Fontes, Jon Ramsey, Jeremy M Polk, Andre Koop, Janna V. Denisova, Andrei B. Belousov

**Affiliations:** 1 Department of Biochemistry and Molecular Biology, University of Kansas Medical Center, Kansas City, Kansas, United States of America; 2 Department of Molecular and Integrative Physiology, University of Kansas Medical Center, Kansas City, Kansas, United States of America; Universidade Federal do ABC, BRAZIL

## Abstract

Pharmacological blockade or genetic knockout of neuronal connexin 36 (Cx36)-containing gap junctions reduces neuronal death caused by ischemia, traumatic brain injury and NMDA receptor (NMDAR)-mediated excitotoxicity. However, whether Cx36 gap junctions contribute to neuronal death via channel-dependent or channel-independent mechanism remains an open question. To address this, we manipulated connexin protein expression via lentiviral transduction of mouse neuronal cortical cultures and analyzed neuronal death twenty-four hours following administration of NMDA (a model of NMDAR excitotoxicity) or oxygen-glucose deprivation (a model of ischemic injury). In cultures prepared from wild-type mice, over-expression and knockdown of Cx36-containing gap junctions augmented and prevented, respectively, neuronal death from NMDAR-mediated excitotoxicity and ischemia. In cultures obtained form from Cx36 knockout mice, re-expression of functional gap junction channels, containing either neuronal Cx36 or non-neuronal Cx43 or Cx31, resulted in increased neuronal death following insult. In contrast, the expression of communication-deficient gap junctions (containing mutated connexins) did not have this effect. Finally, the absence of ethidium bromide uptake in non-transduced wild-type neurons two hours following NMDAR excitotoxicity or ischemia suggested the absence of active endogenous hemichannels in those neurons. Taken together, these results suggest a role for neuronal gap junctions in cell death via a connexin type-independent mechanism that likely relies on channel activities of gap junctional complexes among neurons. A possible contribution of gap junction channel-permeable death signals in neuronal death is discussed.

## Introduction

Gap junctions connect neighboring cells via intercellular channels that allow direct electrical communication and propagation of ions and small molecules between coupled cells [1]. The channels are made of proteins known as connexins, which are encoded in mammals by a family of 21 connexin genes [2]. Connexin 36 (Cx36) is the primary neuronal connexin [3]. In the mammalian CNS, coupling of neurons by gap junctions and the expression of Cx36 rapidly increase (usually during 1–2 hours) following a wide range of neuronal injuries, including ischemia [4–6], traumatic brain injury (TBI) and spinal cord injury [7–9], retinal injury [10], epilepsy [11, 12] and inflammation [13]. Pharmacological and genetic blockade of Cx36-containing gap junctions dramatically reduce neuronal death in animal models of ischemia, TBI and epilepsy [6, 14–17] and prevent NMDA receptor (NMDAR)-mediated excitotoxicity [14, 18, 19]. In addition, group II metabotropic glutamate receptors (mGluR) control the injury-mediated increase in neuronal gap junction coupling and the blockade of these receptors both prevents the up-regulation of neuronal gap junctions and reduces injury-dependent neuronal death [6, 15].

It has been suggested previously [20, 21] that overactivation of glutamate receptors (mainly NMDARs) is an underlying mechanism for the secondary neuronal death following neuronal injury. Based on our studies on neuronal gap junctions, we recently proposed a modified model for the mechanisms of glutamate-dependent excitotoxicity [22, 23]. We speculated that the hyperactivation of NMDARs *per se* is not the main driver of glutamate-dependent neuronal death following injury. Rather, the key determinant for expansion of cell death is the presence and extent of Cx36 gap junctions in the injured CNS regions. In the absence of neuronal gap junctions, NMDAR-mediated neuronal death is restricted to a limited number of neurons. However, in the presence of gap junctions, the expanse of cell death extends to nearly all coupled neurons. In addition, the increase in neuronal gap junction coupling that results from activation of group II mGluRs, is a significant, underlying driver of expansive neuronal death. We proposed that this is a general mechanism for neuronal death during different types of neuronal injury [22, 23]. However, whether the contribution of neuronal gap junctions to cell death is solely dependent upon channel activity, is channel-independent or a combination of both is not known. Stated another way: is the role of Cx36 in neuronal death limited to providing cell-cell fluxes of ions or metabolites through gap junction channels or are there activities specific to this connexin isoform beyond its channel functions? This was addressed in the present study.

## Materials and Methods

### Ethics

The use of animal subjects in these experiments was approved by the University of Kansas Medical Center Animal Care and Use Committee (protocol number 2013–2126). The experiments were conducted in accordance with the National Institutes of Health guidelines. We used two groups of mice: wild-type (WT, C57bl/6) and Cx36 knockout (C57bl/6 background strain). The Cx36 knockout was originally created by Dr. David Paul (Harvard Medical School) [24]. Mice were genotyped as described [18].

### Culture of primary cortical neurons and HeLa cells

Neuronal cultures were prepared as reported previously [19] from the somatosensory cortex obtained from day 16 mouse embryos (either WT or Cx36 knockout; either sex). Pregnant mice were anesthetized with isoflurane before embryos were removed. After disaggregation using papain, neurons were plated on glass coverslips and cultured in Neurobasal medium (Invitrogen, Carlsbad, CA, USA, cat.# 21103), that promotes the development of nearly pure neuronal cultures (containing up to 95% neurons) [25]. In addition, to suppress proliferation of glial cells, cytosine β-D-arabinofuranoside (1 μM) was added to the culture medium on day *in* vitro 2 (DIV2). The Neurobasal medium also was supplemented with B-27 (Invitrogen, cat.# 17504), 0.5 mM L-glutamine and 5 mg/100 ml gentamicin. The medium in cultures was changed twice a week. Lentiviral transduction (see below) was done in neuronal cultures on DIV3-6 (in Cx36 knockout mouse cultures) or DIV10 (in WT mouse cultures). DIV10 WT neurons were chosen for transduction since subsequent neuronal death experiments were conducted at DIV13, i.e., at the time when endogenous neuronal gap junction coupling is maximal in developing neurons [19, 26]. For Cx36 knockout neurons this timing was not essential, therefore, the transduction was done earlier. Forty-eight hours after lentiviral transduction, in some cultures, NMDAR excitotoxicity was performed as we previously described [19], by adding 10 μM NMDA to the culture medium for 30 minutes followed by wash out with the normal Neurobasal medium. In other cultures, oxygen-glucose deprivation (OGD) was conducted as we previously described [6], where the cultures were transferred for 30 minutes from normal conditions (95% air + 5% CO_2_; 10 mM glucose) to OGD conditions (95% N_2_ + 5% CO_2_; no glucose) before returning cells to normal culture conditions. For both NMDA and OGD injuries, the controls included substitution of the incubating medium with the same medium for 30 min.

HeLa cells, obtained from the American Type Culture Collection (ATCC, Manassas, VA; cat.# CCL-2) were cultured in Dulbecco’s Modified Eagle’s medium (Sigma-Aldrich, St. Louis, MO; cat.# D6429) supplemented with 10% fetal bovine serum (Atlanta Biologicals, Lawrenceville, GA; cat.# S11550FBS) and 10 units/ml penicillin/streptomycin (Sigma-Aldrich; cat.# P4333).

In the present study, HeLa cells were used in scrape loading and other experiments in order to characterize the signal permeability and cell membrane localization of virally-transduced wild-type and mutant gap junction channels (see below). These cells were chosen because the cell lines (including HeLa) have been used effectively in these types of experiments [27, 28]. In addition, difficulty in preparation of viable primary neuronal cultures of a very high density and the extreme vulnerability of cultured neurons both preclude the use of neuronal cultures in scrape loading tests.

### Preparation of lentiviral vectors expressing WT connexins

Polymerase chain reaction products of mouse Cx36, connexin 43 (Cx43) and connexin 31 (Cx31) cDNA were created using Q5 High Fidelity DNA Polymerase (New England Biolabs, Ipswich, MA; cat.# M0491S) and the primers listed below: Cx36_F, 5’-TTTGAATTCGCCTCCGGATGCACAGCGATG-3’; Cx36_R, 5’-TAAGCGGCCGCTTCACACATAGGCAGAGTC-3’; Cx43_F, 5’-TTTGAATTCaaagagaggtgcccagacatg-3’; Cx43_R, 5’-ATAGCGGCCGCTTTAAATCTCCAGGTCATC-3’; Cx31_F, 5’-ATCGAATTCACCATGGACTGGAAGAA-3’; Cx31_R, 5’-AGCGGCCGCTCTCTAGCACCTGCTTC-3’. cDNAs for Cx36, Cx43 and Cx31 were obtained from Thermo Scientific (Waltham, MA) corresponding to clone ID’s 5364942, 3673723 and 4983869, respectively, and used as templates in polymerase chain reaction. Amplified products were subcloned into pCMV-SPORT6 (Life Technologies, Grand Island, NY) before final placement into pCDH-MSCV-MCS-EF1-Puro for lentiviral production (Systems Biosciences, Mountain View, CA; cat.# CD710B-1; contains green fluorescent protein {GFP}). A plasmid for production of lentivirus expressing *Cypridina* luciferase (pCDH-LUC) was constructed previously [29], and was included as a control. All pCDH constructs were transformed into Stbl3 cells (Invitrogen; cat.# C737303) and confirmed by sequencing (Genewiz, South Plainfield, NJ). Lentiviral vectors were generated by co-transfection of the pCDH protein expression plasmid with psPAX2 and pMD2.g (Addgene, Cambridge MA; plasmid numbers 12260 and 12259, respectively) [30] for 4 hours in low passage HEK293T cells (Thermo Scientific; cat.# HCL4517). Forty-eight hours later, the supernatant was collected and filtered through a 0.45 μM polyethersulfone membrane. Titers were concentrated by ultracentrifugation at 30,000 x *g* and 15°C for 2 hours and pellets then were resuspended in a small volume of Neurobasal medium. Lentiviral titers were determined by seeding U-937 cells (ATCC; cat.# CRL-1593.2) in 24-well plates at 5 x 10^4^ cells per well on the day of transduction with serial dilutions of the concentrated stock virus. U-937 cells then were incubated for 2 days. Cells expressing GFP were identified on a BD LSR II flow cytometer (BD Biosciences, San Jose, CA) with titers ranging from 5x10^7^ to 2x10^8^ infectious units per mL.

The three WT connexin expressing lentiviruses (derived from pCDH-Cx36, pCDH-Cx43 and pCDH-Cx31) and the control lentivirus (derived from pCDH-LUC) were singly transduced into HeLa cells or neuronal cortical cultures (obtained from Cx36 knockout mice), where their expression was characterized (see Results). For transduction in HeLa cells, a multiplicity of transduction (MOI) of 5 was employed. Two days after transduction, 1μg/mL puromycin (InvivoGen, San Diego, CA; cat.# ant-pr-1) was added to the medium and the cells were cultured for two more weeks. This allowed the selection of HeLa cells stably expressing the corresponding virally-induced protein and GFP (confirmed as described in Results). For neuronal culture transduction, a MOI = 20 was used.

### Preparation of lentiviral vectors expressing mutant Cx36 and Cx43

Using the WT mouse Cx36 lentiviral construct that is described above, three Cx36 mutants were created using a QuikChange Lightning Site-Directed Mutagenesis kit (Agilent Technologies, Santa Clara, CA; cat.#210518). The following primers were used to render two double substitution mutants of either L10A/Q15A or L10P/Q15P in addition to an I22R substitution mutant.: Cx36_L10A-F, 5'-ACCATCTTGGAGAGGGCCCTGGAAGCCGCGGTG-3'; Cx36_L10A-R, 5'-CACCGCGGCTTCCAGGGCCCTCTCCAAGATGGT-3'; Cx36_Q16A-F, 5'-CTGGAAGCCGCGGTGGCCCAGCACTCCACTATGA-3'; Cx36_Q16A-R, 5'-TCATAGTGGAGTGCTGGGCCACCGCGGCTTCCAG-3'; Cx36_L10P-F, 5'-CCATCTTGGAGAGGCCTCTGGAAGCCGCGGTG-3'; Cx36_L10P-R, 5'-CACCGCGGCTTCCAGAGGCCTCTCCAAGATGG-3'; Cx36_Q16P-F, 5'-TGGAAGCCGCGGTGCCTCAGCACTCCACTATG-3'; Cx36_Q16P-R, 5'-CATAGTGGAGTGCTGAGGCACCGCGGCTTCCA-3'; Cx36_I22R-F, 5'-GGTGCAGCAGCACTCCACTATGAGGGGGAGGATCCTG-3'; Cx36_I22R-R, 5'-CAGGATCCTCCCCCTCATAGTGGAGTGCTGCTGCACC-3'.

It has been shown earlier [31], that a single amino acid mutation G21R in Cx43 protein causes formation of gap junction channels that successfully reach cell-cell interfaces between cell pairs, but are communication-deficient. Therefore, a similar mutant was used in the present study. The Cx43 G21R mutant was created using the WT Cx43 lentiviral construct (see above), the same QuikChange kit used for making the Cx36 mutants and the following primers: Cx43_G21R-F, 5'-CTACTCCACGGCCAGAGGGAAGGTGTG-3'; Cx43_G21R-R, 5'-CACACCTTCCCTCTGGCCGTGGAGTAG-3'.

The four mutant connexin lentiviral vectors, pCDH-Cx36(AA) (L10A/Q15A mutation), pCDH-Cx36(PP) (L10P/Q15P mutation), pCDH-Cx36(I22R) and pCDH-Cx43(G21R), were used to generate lentivirus that was singly transduced into HeLa cells (stable transduction; as described above for WT constructs) and into Cx36 knockout mouse neuronal cortical cultures (MOI = 20), where their expression was then characterized as described in Results.

### Preparation of lentiviral vectors expressing Cx36-targeted small hairpin RNA (shRNA)

To study the effects of down-regulation of endogenous Cx36 protein, two shRNAs were designed using Invitrogen’s shRNA designer and blasted against mouse Reference Sequence (RefSeq) database. The shRNAs were cloned into pGreenPuro (Systems Biosciences; cat.# SI505A-1) per the manufacture’s recommendations and using the following oligonucleotides: shRNA1-F, 5’-GATCCGGAGACGGTGTACGATGATGACTTCCTGTCAGATCATCATCGTACACCGTCTCCTTTTTG-3’; shRNA1-R, 5’-AATTCAAAAAGGAGACGGTGTACGATGATGATCTGACAGGAAGTCATCATCGTACACCGTCTCCG-3’; shRNA2-F, 5’-GATCCGCATTTGTGTGGTGCTCAATCCTTCCTGTCAGAGATTGAGCACCACACAAATGCTTTTTG-3’; shRNA2-R, 5’-AATTCAAAAAGCATTTGTGTGGTGCTCAATCTCTGACAGGAAGGATTGAGCACCACACAAATGCG-3’.

shRNA1-Cx36 and shRNA2-Cx36 targeted the Cx36 cDNA positions at 611 and 1270, respectively. A plasmid that enables expression of a shRNA directed against *Cypridina* luciferase (pGreenPuro-shLuc) was constructed previously [29], and was included as a control. Lentiviral constructs were made using the same plasmids (psPAX and pMD2.g) and technical approaches as outlined above for the WT and mutant connexin viruses.

### Immunostaining

Staining procedures were as we described previously [6, 19]. Coverslips containing cultured neurons or HeLa cells were fixed in 4% paraformaldehyde for 15 minutes and then washed three times in phosphate buffered saline (PBS; 0.01 mol/L). Nonspecific background was blocked by overlaying 2% bovine serum albumin (Fisher-Scientific, Waltham, MA; cat.# BP1600-100) + 0.2% Triton X-100 in PBS for 30 minutes. The cells then were incubated in the presence of a primary antibody: rabbit anti-NeuN (conjugated to Alexa Fluor 350; Bioss, Woburn, MA; cat.# bs-1613R-A350; 1:100 in PBS; 1 hr), rabbit anti-Cx36 (Invitrogen; cat.# 51–6300; 1:100 in PBS; overnight at 4°C), rabbit anti-Cx43 (Life Technologies; cat.# 71–0700; 1:100 in PBS; overnight at 4°C) or rabbit anti-Cx31 (Invitrogen; cat.# 36–5100; 1:100 in PBS; overnight at 4°C). After washing (3 times), the cells pre-treated with a noncojugated primary antibody were incubated with the goat anti-rabbit Alexa Fluor 555 antibody (Abcam, Cambridge, MA; cat.# 150078; 1:500 in PBS for 1 hour at room temperature). After the final wash (3 times), cells were covered with Vectashield mounting medium (Vector Laboratories, Burlingame, CA; cat.# H-1000) and coverslips were mounted on microscope slides.

For the analysis of co-expression of GFP and NeuN (a neuron-specific nuclear protein) in neuronal cultures, fluorescent signals were visualized on a Nikon Eclipse 80i using FITC and Texas Red filters, respectively. Images were captured on a Photometrics ES2 digital camera using OpenLab software (Improvision, Lexington, MA, USA). For the analysis of expression of Cx36, Cx43 or Cx31 in stably-transduced HeLa cells, laser scanning confocal Nikon Eclipse C1 Plus microscope, Nikon 60X oil objective and Nikon EZ-C1 software were used. In each group, the analysis was done in three coverslips obtained from independent cell culture preparations or HeLa cell lines. The antibodies that were used for analysis of expression of WT connexins also recognized mutant proteins (see Results). Negative controls included staining for the secondary antibody without preincubation with the primary antibody (12 coverslips total).

### Western blots

Experiments were performed as reported in detail [19, 26]. Briefly, cultured cells were homogenized in a lysis buffer and total protein was determined using the Bio-Rad DC protein assay method. Fifty μg of protein was loaded in each lane, transferred to 0.45 μm Polyvinylidene difluoride membrane and processed with a blocking solution and antibodies. The primary connexin antibodies (the same as in immunostaining experiments) were used in the following concentrations: anti-Cx36, 0.5 μg/ml; anti-Cx43, 0.2 μg/ml; anti-Cx31, 0.5 μg/ml. These antibodies also were successfully used for analysis of expression of mutant proteins (see Results). Signals were visualized with horseradish peroxidase conjugated anti-rabbit antibody (1:10,000, Life Technologies, cat.# G21234). The signals were enhanced using ECL detection reagents (Amersham Biosciences, Piscataway, NJ, USA). Tubulin levels per unit of total protein did not vary significantly among samples used in this study. Each experiment was done in triplicate.

### Methyl thiazolyl tetrazolium (MTT) assay

Neuronal viability in cultures was quantitatively evaluated by MTT assay. Experiments were performed as reported in detail [6, 19]. Cultures were raised in 24-well plates. For analysis of the effects of lentiviral transductions on neuronal cell survival, Cx36 knockout cultures were transduced on DIV6 and MTT tests were done 3 days later. For analysis of neuronal death following NMDA or OGD injuries in transduced neuronal cultures, the cultures were transduced on DIV6 (Cx36 knockout) or DIV10 (WT), the injuries were induced 48 hours post-transduction, followed by MTT tests at 24 hours post-injury. In all MTT tests, neurons were incubated with MTT (MTT cell Viability Assay Kit; Biotium, Inc., Hayward, CA, USA; 40 μM, 400 μL per well) at 37°C for 4 hours. Then the medium was carefully aspirated and 400 μL of DMSO per well was added to dissolve the blue formazan product. To measure the absorbance, 200 μL of the medium from each well in 24-well plate were transferred into an independent well in a 96-well plate. The values of absorbance at 570 nm were measured using a microplate reader (μQuant, BioTek, Winooski, VT, USA). Further, as indicated above, cultures that are raised in Neurobasal medium contain mostly neurons (up to 95%). However, to control specifically for neuronal cell death, in a separate group of cultures (*n* = 6) 500 μM glutamate was added to the cell culture medium and was present in the medium for 24 hours prior to conducting MTT assay. This killed all neurons, but did not affect glial cell survival. The absorbance in these purified glial cultures was measured, averaged, and the result was subtracted from the individual absorbance data in neuronal culture groups so that the final result would represent only neuronal death/survival. Finally, the absorbance results in experimental groups were normalized to control groups.

### Scrape-loading dye transfer assay

Experiments were performed as reported [27]. Tests were done in HeLa cells stably-transduced with one of the WT or mutant connexin lentiviral vectors or the control vector (pCDH-LUC) (for the rationale of using HeLa cells and not neurons in these experiments see above). Cells were seeded 24 hours before the scrape assay at a density of 1.25 x 10^5^ cells per well in an 8-well chamber slide. Before the test, the incubating medium was replaced with the medium containing Neurobiotin (0.5 mg/mL; Vector Laboratories; cat.# SP-1120; MW323; gap junction/hemichannel-permeable dye) and dextran Alexa Fluor 594 (0.5 mg/mL; Invitrogen, cat.# D22913; MW10,000; gap junction/hemichannel-impermeable dye). Cells were scrape-loaded using a sterile scalpel by drawing 2 cuts from corner to corner to form an “X” across the well. The cells were incubated in the medium for 5 minutes at room temperature to allow the dyes to infiltrate the cut cells. The cells then were washed (3 times with PBS, 0.01 mol/L) and fixed in 4% paraformaldehyde for 30 minutes in the dark. The cells were washed again (3 times), permeabilized with PBS + 1% triton-X for 30 minutes, washed (3 times) and the nonspecific binding was blocked with PBS + 0.1% Tween-20 + 10% fetal bovine serum for another 30 minutes. After washing (3 times with PBS), a probe for Neurobiotin, AMCA Avidin-D (Vector Laboratories; cat.# A-2008), was added at 1:500 in PBS + 0.1% Tween-20 + 0.1% bovine serum albumin for 30 minutes. Cells were washed (5 times), the chamber slide was removed and the slide was mounted with Vectashield mounting medium. Fluorescent images of the scrape-loaded cells were captured with a Photometrics ES2 digital camera on a Nikon Eclipse 80i microscope using OpenLab software. A bright-field image and three fluorescent images were captured from the same microscope field using UV-2E/C, FITC, and Texas Red filters to measure DAPI, FITC, and Texas Red emission for AMCA Avidin-D, GFP and dextran Alexa Fluor 594, respectively. Each experiment was done in triplicate. The analysis of gap junction channel permeability was made based on the number of cells containing Neurobiotin (indicating both initially loaded cells and the cells that obtained the dye via gap junction channels), excluding the cells containing dextran Alexa Fluor 594 (indicating only initially loaded cells).

### Dye uptake

We utilized ethidium bromide (EtdBr) uptake that was previously used to measure the activity of hemichannels in cultured neurons [32, 33]. Experiments were performed in purified neuronal cortical cultures (see above) prepared from WT mice and plated on glass coverslips. On DIV10, the cultures were subjected for 30 minutes to NMDA (10 μM) or OGD. Two hours later, the cultures were exposed to 0.5 μM EtdBr (Fisher Scientific; cat.# BP1302-10) for 10 min at 37°C followed by wash out with incubating medium. To visualize EtdBr uptake, the cells were examined using Nikon Eclipse 80i microscope with a Nikon Super Fluor 20X objective, Texas Red filter and OpenLab software. The dye uptake analysis was done in neurons; the percentage of EtdBr-positive neurons per microscope field was calculated. Neurons had the characteristic "phase bright" round or oval cell bodies, small size (~10–15 μm), multiple tiny processes and were easily distinguished from astrocytes, which had a bigger size (50–100 μm), exhibited flattened morphology and did not have a clear-cut cell body and processes (in the bright field, they often could not be differentiated from the cellular debris). For each experimental condition, three coverslips with cultures were analyzed, with 10 microscopic fields per coverslip chosen randomly and the data were averaged.

### Statistical analysis

Data were analyzed using the two-tailed Student's *t*-test (paired, when possible) or ANOVA with post hoc Tukey and InStat software (GraphPad Software, San Diego, CA, USA). Data are reported as mean ± SEM. The number of samples indicated represents the number of plate wells/coverslips with cells obtained at least from three independent cell culture preparations, i.e., in case of neuronal cultures—from three dams; in case of HeLa cells—from three seedings. At the end of all experiments, the data for each experimental group were combined. The analyses were performed blinded to the treatment conditions.

## Results

### Characterization of connexin lentiviral vectors

In the present study, we addressed whether: (1) changes in the amount of neuronal gap junctions directly affect neuronal death and survival and (2) neuronal death related to gap junctions depends upon their channel activity. We prepared three lentiviral plasmids (pCDH-Cx36, pCDH-Cx43 and pCDH-Cx31; see Materials and Methods) to express WT neuronal Cx36 or non neuronal Cx43 (found in astrocytes; [3]) or Cx31 (found in skin, placenta, kidney and testes, but not the CNS; [34]). We also produced four mutant connexin lentiviral vectors: pCDH-Cx36(AA) (L10A/Q15A double substitution mutation in Cx36), pCDH-Cx36(PP) (L10P/Q15P double substitution mutation in Cx36), pCDH-Cx36(I22R) (single amino acid mutation I22R in Cx36) and pCDH-Cx43(G21R) (single amino acid mutation G21R in Cx43). In addition, two Cx36-targeted shRNA-expressing vectors (shRNA1-Cx36 and shRNA2-Cx36) were prepared. Appropriate expression and formation of gap junctional complexes from WT and mutant connexin lentiviruses were characterized, as described below, in neuronal cortical cultures obtained from Cx36 knockout mice and in stably-transduced HeLa cells. Cx36 shRNA effectiveness in knockdown was characterized in WT mouse neuronal cortical cultures. Lentiviral vectors enabling expression of a *Cypridina* luciferase gene (pCDH-LUC) or shRNA directed against *Cypridina* luciferase (shRNA-LUC) were used as controls.

**Fig 1:**
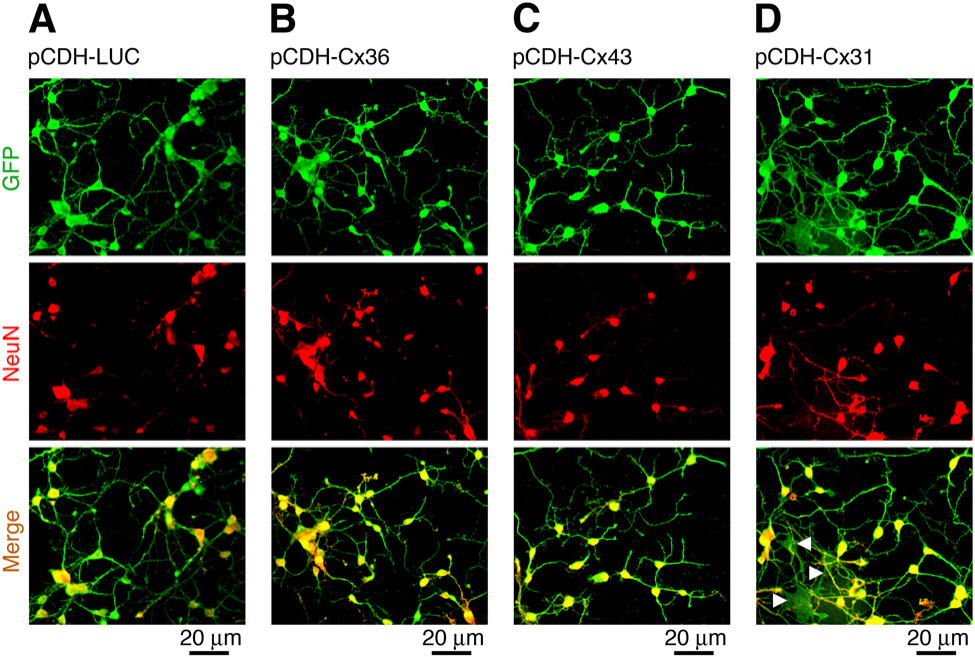
Lentivirus expressing connexins effectively transduce neurons. Data from immunostaining experiments in neuronal cortical cultures obtained from Cx36 knockout mice are presented. Cells were transduced on DIV3 with pCDH-LUC (control vector; **A**), pCDH-Cx36 (**B**), pCDH-Cx43 (***C***) and pCDH-Cx31 (**D**) and the staining analysis was done 48 hours later. GFP (transduction marker), NeuN (neuronal marker) and merged images are shown. GFP and NeuN co-localization indicates the infected neurons. Transduction efficiency was >90%. Glial cells (presumably astrocytes), that were present in small amounts in these neuronal cultures (see Materials and Methods), also showed lentiviral transductions (denoted by arrowheads in the merged image in **D**). Images are representative of three experiments in independent cell cultures and transductions.

**Fig 2:**
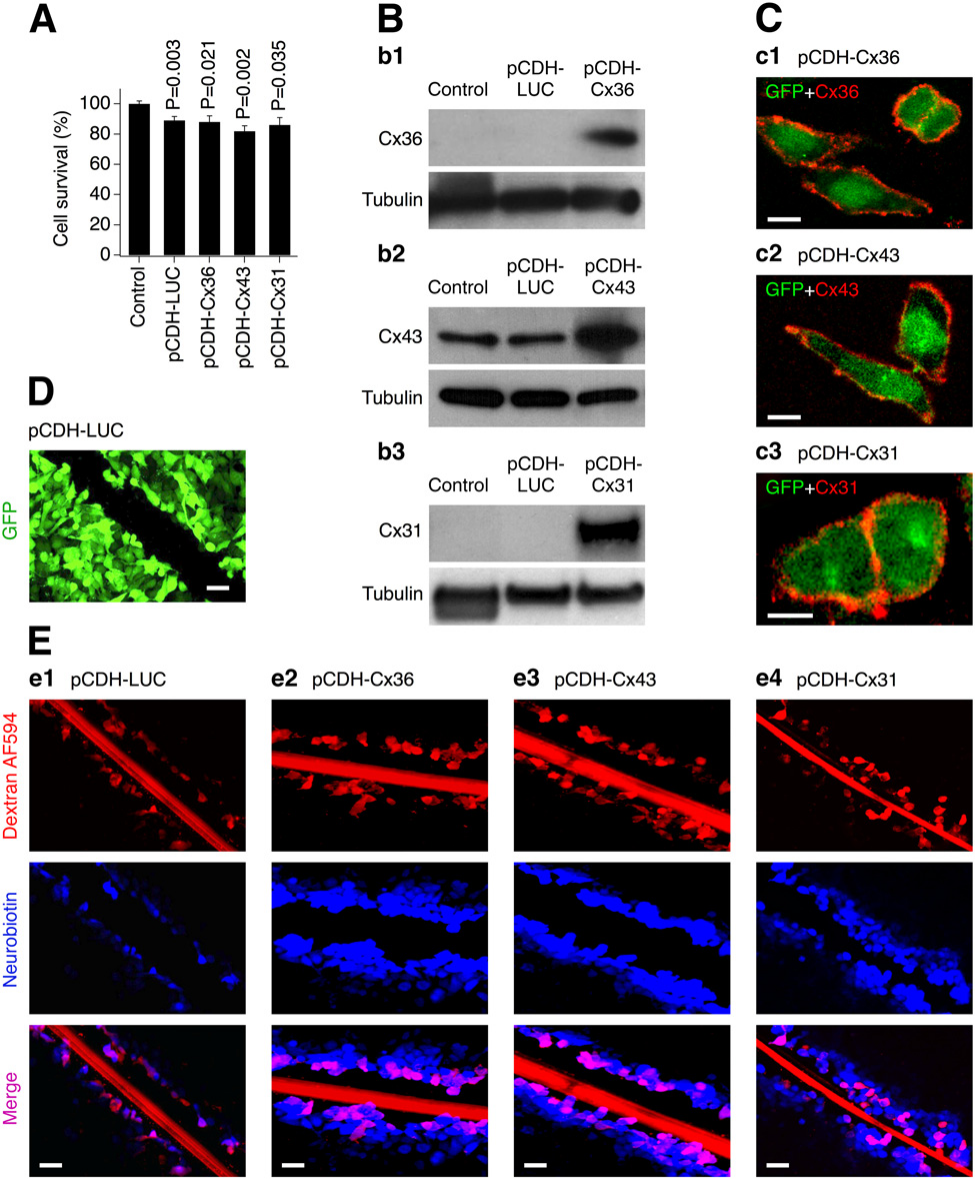
Expression of connexins from lentivirus induces functional gap junction channels. Data from MTT assay (**A**), western blot (**B**), immunostaining (**C**) and scrape-loading dye transfer (**D, E**) experiments in transduced Cx36 knockout mouse neuronal cortical cultures (**A**, **B**) and stably-transduced HeLa cells (**C-E**) are shown. **A**, Lentiviral transductions reduce neuronal survival over a three day period (statistical analysis: Student's *t*-test, shown relative to non-infected {Control} cultures; *n* = 5–10 per group; mean ± SEM). However, no statistical difference in neuronal survival between the control lentivirus and each of the other transductions was detected (P>0.05, not shown; Student's *t*-test). **B**, Lentiviral transductions express Cx36 (**b1**), Cx43 (**b2**) and Cx31 (**b3**) (these neuronal cultures have contained small amount of astrocytes that explains some background expression of Cx43). In **A**, **B**, cultures were infected on DIV6 and the analyses were done three (**A**) and two (**B**) days post-transduction. **C**, Connexins are expressed in the cell’s plasma membrane (**c1–c3**). Shown are superimposed confocal images of GFP (green; denotes the transduced cells) and staining for the corresponding connexin (red). **D**, This image illustrates that HeLa cells stably-transduced with pCDH-LUC all express GFP (the same was observed for all three connexin-expressing lentiviruses, but is not shown here). The image was taken from the same microscope field as in **e1** (see below). **E**, Cells transduced with lentivirus expressing luciferase (negative control) do not pass a gap junction-permeable dye, Neurobiotin (**e1**), however, the cells transduced with any of the three connexin-expressing lentiviruses do pass Neurobiotin (**e2–e4**). None of the experimental cell groups passes Alexa Fluor 594-tagged dextran (Dextran AF594; gap junction-impermeable dye). Individual and merged images are shown. Thick red line visible across the field is the mark left by the scalpel blade used to scratch cell monolayer. Wounded cells were allowed to absorb both dyes for 5 minutes and were subsequently washed out. In **B-E**, the images are representative for three independent experiments. Calibration bar: 20 μm (**C**) and 50 μm (**D**, **E**).

In Cx36 knockout mouse neuronal cultures transduced with lentivirus expressing WT connexins (or control lentivirus) for each of the vectors the transduction efficiency in neurons was >90%, measured 48 hours post-transduction by expression of GFP (the transduction marker) and NeuN (a neuron-specific nuclear protein) (Fig 1). Though these were purified neuronal cultures, some glial cells (presumably astrocytes) present in small amounts also were transduced (Fig 1D). MTT assay revealed that all transductions reduced neuronal survival slightly over a three day period, compared to non-transduced cultures. However, no statistical difference in neuronal survival was observed when comparing cells transduced with either the control or connexin-expressing lentiviruses (Fig 2A). Therefore, in all of the following experiments with analysis of neuronal death in transduced cultures, data were normalized to the control virus transduction. Western blotting showed expression of the expected proteins 48 hours post-transduction of neuronal cultures (Fig 2B) and in stably-transduced HeLa cell lines (not shown). In addition, immunostaining and confocal microscopic analysis of the stable HeLa cell lines revealed that the corresponding connexins were present at cell plasma membranes: they were commonly seen in cell membrane appositions (Fig 2C: 2c1 and 2c3) and also in membranes of single cells (Fig 2C: 2c1 and 2c2).

To determine if the transgene-expressed connexins formed functional gap junctional complexes, scrape-loading dye transfer assays were performed in stably-transduced HeLa cell lines (Fig 2D and 2E). Essentially all HeLa cells showed GFP labeling, indicating every cell was transduced (Fig 2D). Cells were scrape-loaded with Neurobiotin (a gap junction/hemichannel-permeable dye) and dextran Alexa Fluor 594 (a gap junction/hemichannel-impermeable dye). The number of cells that contained Neurobiotin, but did not contain dextran Alexa Fluor 594 was analyzed. The average number Neurobiotin-positive cells per microscope field was 5.0±1.6 (pCDH-LUC), 83.0±5.2 (pCDH-Cx36; P = 0.0001), 93.3±13.9 (pCDH-Cx43; P = 0.003) and 81.0±4.6 (pCDH-Cx31; P = 0.0001) (statistical significance is shown relative to the pCDH-LUC group; two-tailed unpaired Student's *t*-test; *n* = 3 in each group). This showed that the cells transduced with the control vector do not pass Neurobiotin, since they lack gap junctional coupling. However, the cells expressing any of the three WT connexin proteins (Cx36, Cx43 or Cx31) demonstrated intercellular dye transfer. In these cultures, Neurobiotin was observed both in cells immediately proximal to the scrape (i.e., in damaged cells) and in some of their close neighbors. None of the cultures showed Neurobiotin labeling on the periphery of the cell monolayer. These data support the presence of functional gap junction channels, assembled from the ectopically expressed connexin proteins.

**Fig 3:**
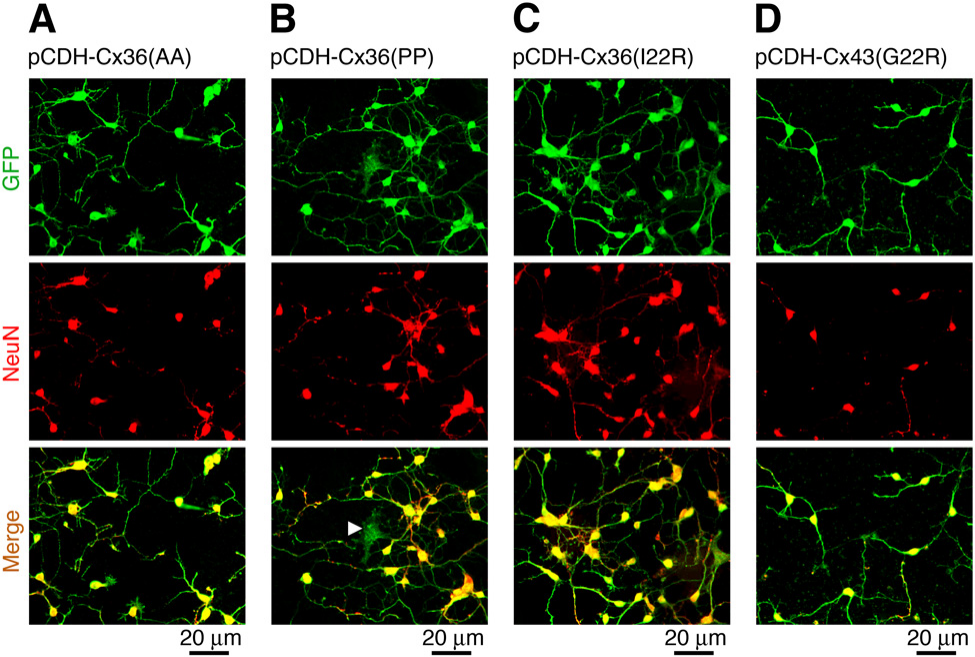
Lentivirus expressing mutated connexins effectively transduce neurons. Data from immunostaining experiments in neuronal cortical cultures obtained from Cx36 knockout mice are presented. Cells were transduced on DIV3 with pCDH-Cx36(AA), which has L10A and Q15A substitutions (**A**), pCDH-Cx36(PP), which has L10P and Q15P substitutions (**B**), pCDH-Cx36(I22R) (**C**) and pCDH-Cx43(G22R) (**D**). Immunostaining was performed 48 hours after transduction. GFP and NeuN co-localization indicates the infected neurons. Transduction efficiency was >90%. Arrowhead in the merged image in **B** denotes a virally transduced glial cell. Images are representative of three experiments in independent cell cultures and transductions.

**Fig 4:**
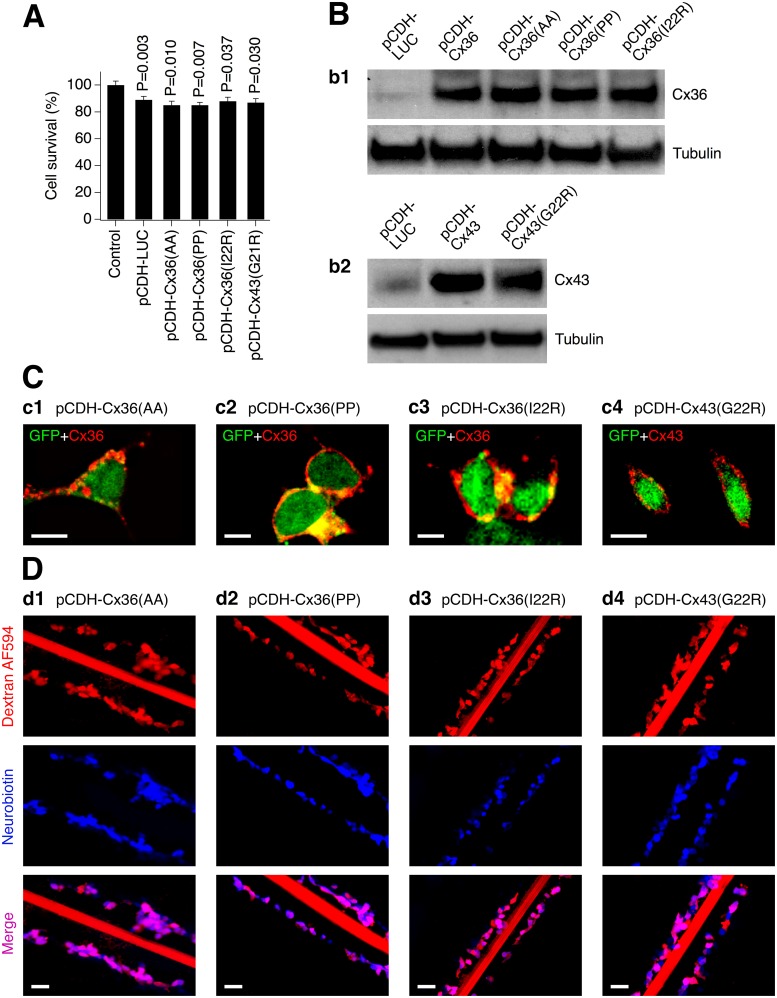
Expression of connexins with amino acid substitutions results in formation of communication-deficient gap junction channels. Data from MTT assay (**A**), western blot (**B**), immunostaining (**C**) and scrape-loading dye transfer (**D**) experiments in transduced Cx36 knockout mouse neuronal cortical cultures (**A**, **B**) and stably-transduced HeLa cells (**C, D**) are shown. **A**, Lentiviral transductions reduced neuronal survival over a three day period (statistical analysis: Student's *t*-test, shown relative to non-infected {Control} cultures; *n* = 4 per group; mean ± SEM). However, no statistical difference in neuronal survival between the control lentivirus and each of the other transductions was detected (P>0.05, not shown; Student's *t*-test). **B**, **C**, Lentiviral transductions induce mutant Cx36 and Cx43 proteins (**B**) that are expressed in the cell’s plasma membrane (**C**). The mutant connexins are recognized by the WT connexin antibodies. **D**, Cells expressing mutant connexin do not pass Neurobiotin. In all experiments, cell transduction conditions, timing, analyses, antibodies (for Cx36 and Cx43) and image presentations are as in Fig 2. In **B-D**, the images are representative of three independent experiments.

Next we characterized the cells expressing Cx36 (L10A/Q15A, L10P/Q15P or I22R) or Cx43 (G21R) containing amino acid substitutions. As with the WT proteins, each of the four mutant connexin expressing lentiviruses effectively transduced neurons in Cx36 knockout neuronal cortical cultures (>90% transduction efficiency; Fig 3). The transductions reduced neuronal survival during a three day period, however, there was no statistical difference in neuronal survival between cultures transduced with the control and mutant connexin lentiviruses (Fig 4A). Western blots showed expression of the mutant connexins in neuronal cultures (Fig 4B) and their stable expression in HeLa cells (not shown) (the amino acid substitutions did not affect antibody recognition of the proteins). In stably-transduced HeLa cells, the mutant connexins successfully reached the plasma membrane (Fig 4C). In scrape scrape-loading tests, the number Neurobiotin-positive, but Alexa Fluor 594-negative cells per microscope field was 2.3±0.7 for pCDH-Cx36(AA) cultures, 3.3±1.2 for pCDH-Cx36(PP), 2.3±1.9 for pCDH-Cx36(I22R) and 3.3±0.9 for pCDH-Cx43(G21R) group (Fig 4D). None of these values was statistically different from that of the control group, i.e., transduced with pCDH-LUC (5.0±1.6; analyzed using two-tailed unpaired Student's *t*-test; *n* = 3 in each group). The data indicate that the mutant gap junction channels are impermeable to Neurobiotin and therefore do not support gap junctional communication between cells.

**Fig 5:**
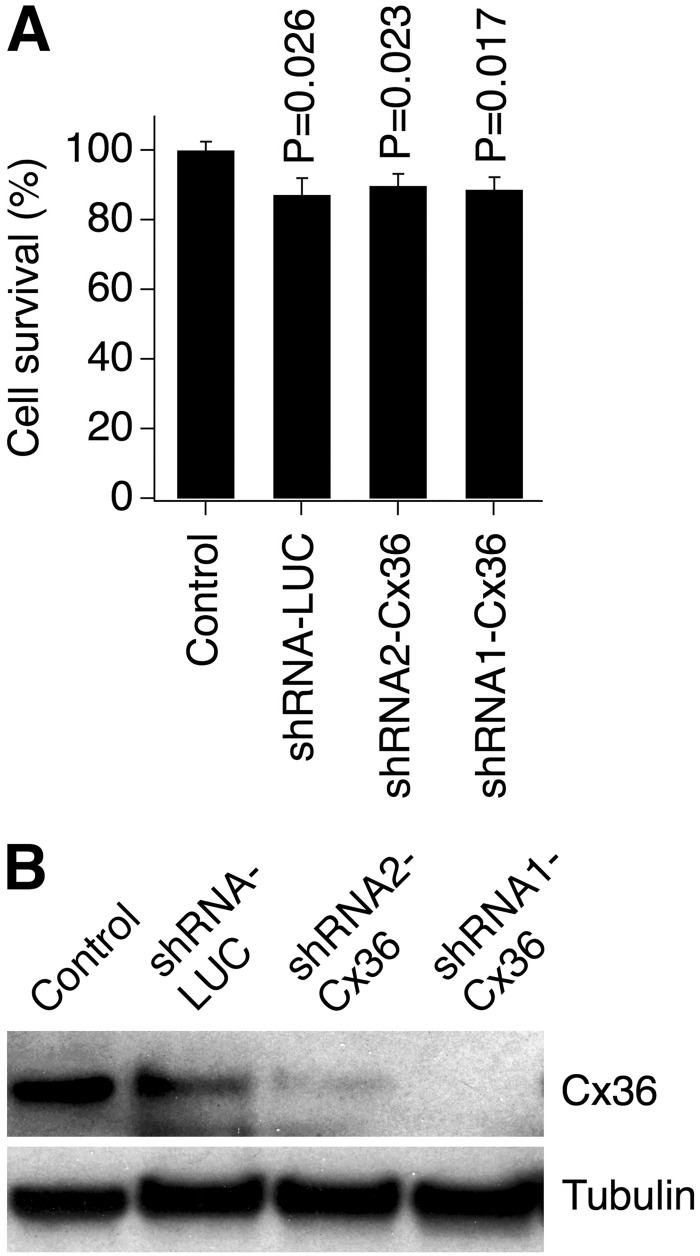
Lentivirally expressed shRNAs targeting Cx36 effectively reduce Cx36 protein levels. Data from MTT assay (**A**) and western blot (**B**) experiments in neuronal cortical cultures obtained from WT mice are shown. Cultures were infected on DIV10 and the analyses were done three days post-transduction. **A**, Lentiviral transductions slightly reduce neuronal survival. Statistical analysis: Student's *t*-test, shown relative to non-infected (Control) cultures; *n* = 7 per group; mean ± SEM. **B**, Transduction with two unique shRNAs directed against Cx36 (shRNA1 and shRNA2) effectively reduced Cx36 protein levels. The image is representative for three independent experiments.

Finally, using WT mouse neuronal cortical cultures we tested two shRNA-Cx36 vectors. As was the case for lentiviruses expressing the connexin proteins, the transduction efficiency for both shRNA vectors was >90% (measured 48 hours post-transduction; data not shown). The transductions induced only moderate reduction in neuronal survival over a three day period (Fig 5A), and both shRNA-Cx36 vectors effectively suppressed the expression of endogenous Cx36 protein (Fig 5B).

### Changes in the amount of neuronal gap junctions directly affect neuronal death and survival

**Fig 6:**
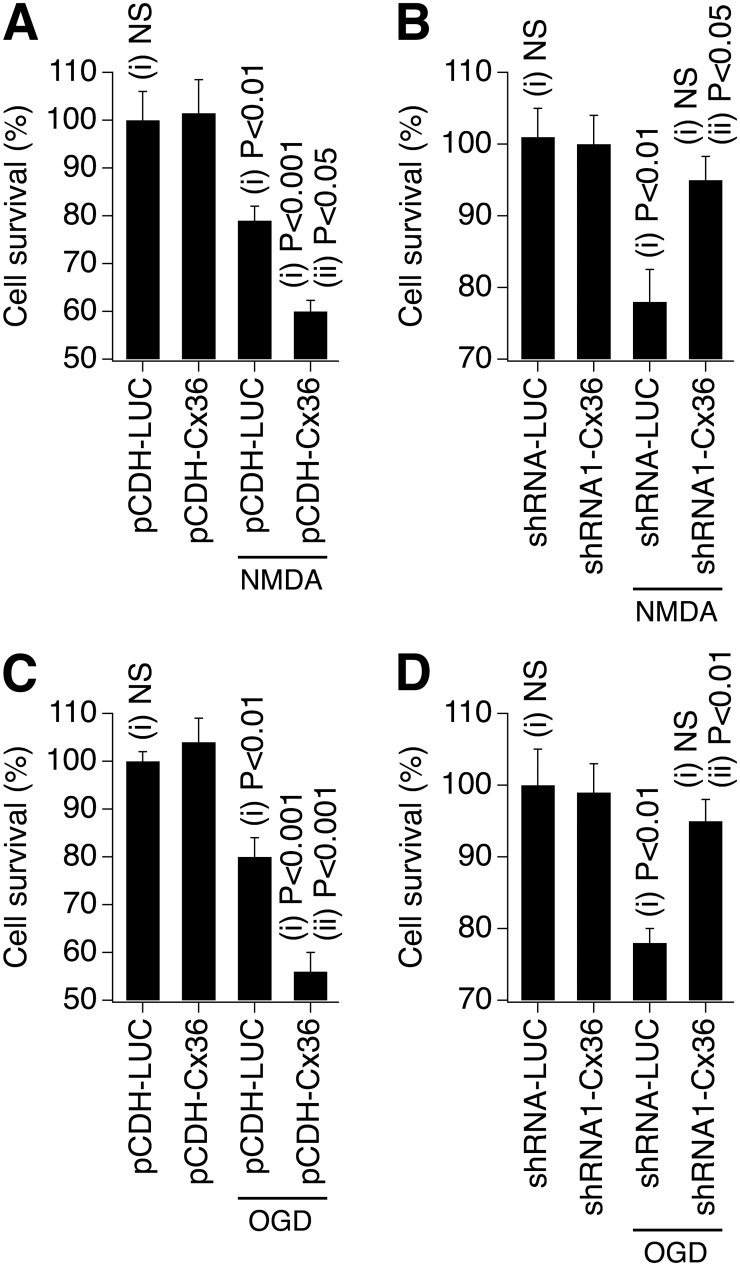
Changes in Cx36 expression affect neuronal death/survival. ***A-D***, Statistical data from MTT assay experiments with the use of NMDAR-excitotoxicity (**A**, **B**) and OGD (**C, D**) in neuronal cortical cultures obtained from WT mice are shown. Cultures were singly transduced with the indicated lentivirus on DIV10; NMDA (10 μM, 30 min) or OGD (30 min) injuries were conducted 48 hours post-transduction; neuronal death analysis was done 24 hours post-injury. Increase (**A**, **C**) and decrease (**B, D**) in the expression of Cx36, respectively, augments and reduces the amount of injury-mediated neuronal death. Note that NMDA treatment and OGD both induce neuronal death and this death is significantly augmented by overexpression of Cx36 (pCDH-Cx36 plus NMDA or OGD in **A** and **C**) and is prevented by knockdown of Cx36 (shRNA1-Cx36 plus NMDA or OGD in **B** and **D**). Statistical analysis: ANOVA with post hoc Tukey; in **A**, **C**, shown relative to (i) pCDH-Cx36 and (ii) pCDH-LUC plus injury (NMDA or OGD); in **B, D**, shown relative to (i) shRNA1-Cx36 and (ii) shRNA-LUC plus injury (NMDA or OGD); in all figures, *n* = 8–12 per group; mean ± SEM; NS, non-significant. Data for one of the shRNA-Cx36 constructs are shown (shRNA1); similar results were obtained for shRNA2 (not shown).

A critical role for Cx36-containing gap junctions in NMDAR-dependent excitotoxicity and injury-mediated neuronal death was suggested previously [22, 23]. However, whether Cx36 gap junctions directly determine neuronal death was not known. Therefore, we tested whether the level of NMDAR-mediated excitotoxicity is proportional to the amount of connexin protein in the cell and the extent of neuronal gap junction coupling. Experiments were performed in WT mouse somatosensory cortical neuronal cultures. On DIV10, cultures were singly transduced with lentivirus that causes either Cx36 over-expression (pCDH-Cx36) or knockdown (shRNA1-Cx36 and shRNA2-Cx36) or transduced with the control lentivirus (pCDH-LUC as control for pCDH-Cx36, and shRNA-LUC as control for shRNA-Cx36 vectors). Forty-eight hours after transduction, 10 μM NMDA was added to the cultures for 30 minutes and then washed out. Twenty-four hours post-NMDA treatment, significant neuronal death was observed in control cultures (transduced with pCDH-LUC or shRNA-LUC) as detected using MTT assay (Fig 6A and 6B). This is consistent with previous reports that NMDAR excitotoxicity is readily detected in DIV12-15 neuronal cultures, i.e., during the period where the transient, developmental increase in the expression of Cx36 gap junctions reaches its peak [18, 19]. NMDAR-mediated neuronal death was significantly increased in pCDH-Cx36-transduced cultures (Fig 6A), which exhibited higher levels of Cx36 expression (Fig 2). Conversely, NMDAR-mediated neuronal death was dramatically reduced in cultures where Cx36 was knocked down by either of two different shRNAs (Fig 6B).

Essentially identical results were obtained in cultures subjected to ischemic injury with a 30-minute OGD (the analysis was done in DIV13 cultures, 3 days post-transduction, including 24 hours post-OGD; Fig 6C and 6D). Together, these findings indicate that neuronal death following injury is directly influenced by the level of Cx36 gap junction coupling between neighboring cells.

### Death of neurons related to gap junctions is channel-dependent

Currently there are two, not necessarily mutually exclusive mechanisms proposed to explain the contribution of gap junctions to cell death: (i) via propagation of gap junction-permeable death signals between the coupled cells [28, 35, 36] and (ii) via channel-independent mechanisms that invoke communication of connexin proteins with intracellular death signaling pathways (reviewed in [37]). Both proposed mechanisms are largely based on experiments with non-neuronal gap junctions. Here we tested whether the contribution of neuronal Cx36 gap junctions in neuronal death is channel-dependent or independent.

**Fig 7:**
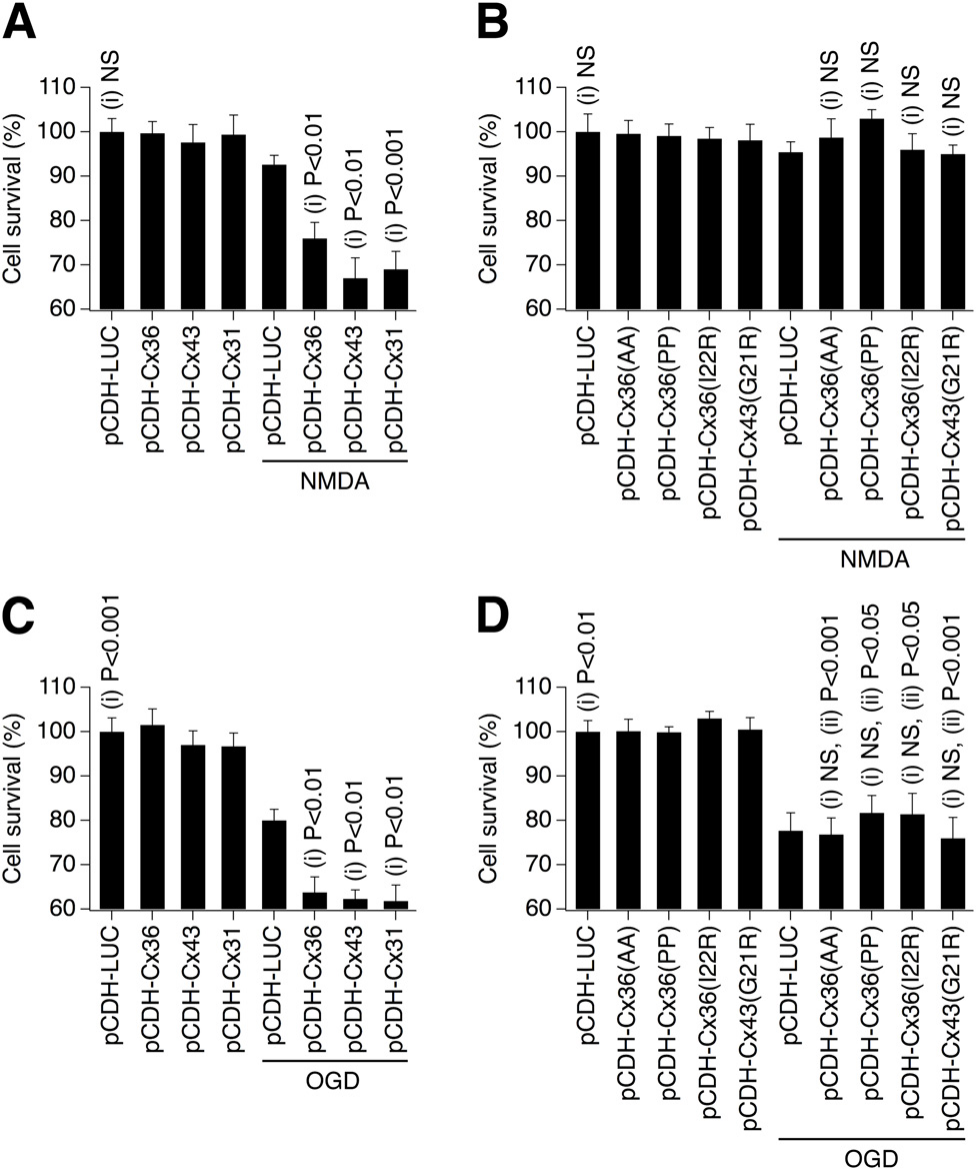
The role of gap junctions in neuronal death is connexin type-independent and requires channel activity. **A-D**, Statistical data from MTT assay experiments with the use of NMDAR-excitotoxicity (**A**, **B**) and OGD (**C**, **D**) in neuronal cortical cultures obtained from Cx36 knockout mice are shown. Cultures were transduced with the indicated lentiviruses on DIV6; NMDA and OGD injuries were conducted 48 hours post-transduction; neuronal death analysis was performed 24 hours post-injury. Functional gap junctions made from neuronal and non-neuronal connexins all support neuronal death (**A**, **C**). Gap junction channels that are communication-deficient do not support neuronal death (**B**, **D**). Statistical analysis: ANOVA with post hoc Tukey; shown relative to (i) pCDH-LUC plus injury (NMDA or OGD) and (ii) pCDH-LUC (without injury); in all figures, *n* = 8–12 per group; mean ± SEM.

The conductance of ions or small molecules by gap junctions so far measured is roughly within one order of magnitude [38], though more significant differences have been reported for larger permeants and some metabolites, with selective permeability based upon charge and size [39, 40]. It is unlikely, though not impossible, that gap junctions resulting from connexins from distinct subfamilies, with significant amino acid differences, would participate in common protein-protein interactions and signaling pathways. Therefore, if multiple, disparate gap junctions support neuronal death in the same cell death paradigm, the most likely mechanism is channel function, where the molecule or ion “death signal” has sufficient permeability through the tested gap junctions. In our experiments, we used neuronal cell transductions with lentivirus expressing neuronal Cx36 or non neuronal Cx43 or Cx31, which belong to three distinct connexin subfamilies with significant differences in amino acid composition in their intracellular domains [41]. To isolate the effects of transduced gap junctions from the contribution of endogenous gap junctions, the experiments were conducted in neuronal cortical cultures prepared from Cx36 knockout mice. On DIV6, cultures were singly transduced with lentivirus expressing WT Cx36, Cx43, Cx31 or *Cypridina* luciferase (control). As demonstrated in control tests (Fig 2B,2C and 2E), all WT connexin lentiviruses resulted in expression of the corresponding connexins and functional gap junctions. Forty-eight hours after transduction, one group of cultures was treated for 30 minutes with 10 μM NMDA followed by wash out. Another group was subjected to OGD for 30 minutes. Twenty-four hours later, MTT assay was conducted. No neuronal death was observed in NMDA-treated cultures that were infected with the control vector (pCDH-LUC; Fig 7A). This supports the previous observations that NMDAR-mediated excitotoxicity is substantially reduced (or absent) in the absence of neuronal gap junction coupling [14, 18, 19]. However, significant NMDAR-dependent neuronal death was detected in cultures expressing either neuronal (Cx36-) or non-neuronal (Cx43- or Cx31-containing) gap junctions (Fig 7A). Following OGD, some neuronal death was detected in the control, but it was augmented in cultures expressing the various WT connexin channels (Fig 7C). Together, these data suggest that the role of gap junctions in neuronal death is connexin type-independent and point to channel function as the primary mechanism for the contribution of gap junctions to neuronal death.

Second, we tested the prediction that communication-deficient gap junction channels cannot support neuronal death. Cx36 knockout mouse neuronal cortical cultures were singly transduced with a lentivirus expressing one of four mutated connexins: pCDH-Cx36(AA), pCDH-Cx36(PP), pCDH-Cx36(I22R) and pCDH-Cx43(G21R), that induce dye-impermeable (i.e., communication-deficient) gap junctions despite the fact they are present in the cell membrane (Fig 4C and 4D). Transductions were performed in cultures on DIV6. Forty-eight hours after transduction, 30-minute NMDA (10 μM) or OGD treatments were conducted followed by MTT assay after an additional 24 hours. Cultures expressing mutant connexins showed neither detectable NMDAR excitotoxicity (Fig 7B) no any additional OGD-induced neuronal death, as compared to pCDH-LUC-infected cultures (Fig 7D). Together, these data suggest that a patent, conducting channel likely is necessary for neuronal gap junctions to induce cell death following injury.

### Are hemichannels involved?

It has been reported that open hemichannels may play a role in mediating neuronal cell death. For example, inflammation-like conditions open Cx43 hemichannels in astrocytes that activates neuronal hemichannels and promotes NMDAR-mediated excitotoxicity in neurons [32]. In addition, exposure of cells to amyloid-β peptide (a model of conditions related to Alzheimer’s disease) also promotes neuronal death via consecutive activation of glial and then neuronal hemichannels [33]. Using EtdBr uptake as an assay for the presence and activity of hemichannels, we tested the possibility that neuronal hemichannels open and contribute to neuronal death caused by NMDA receptor excitotoxicity and ischemia.

**Fig 8:**
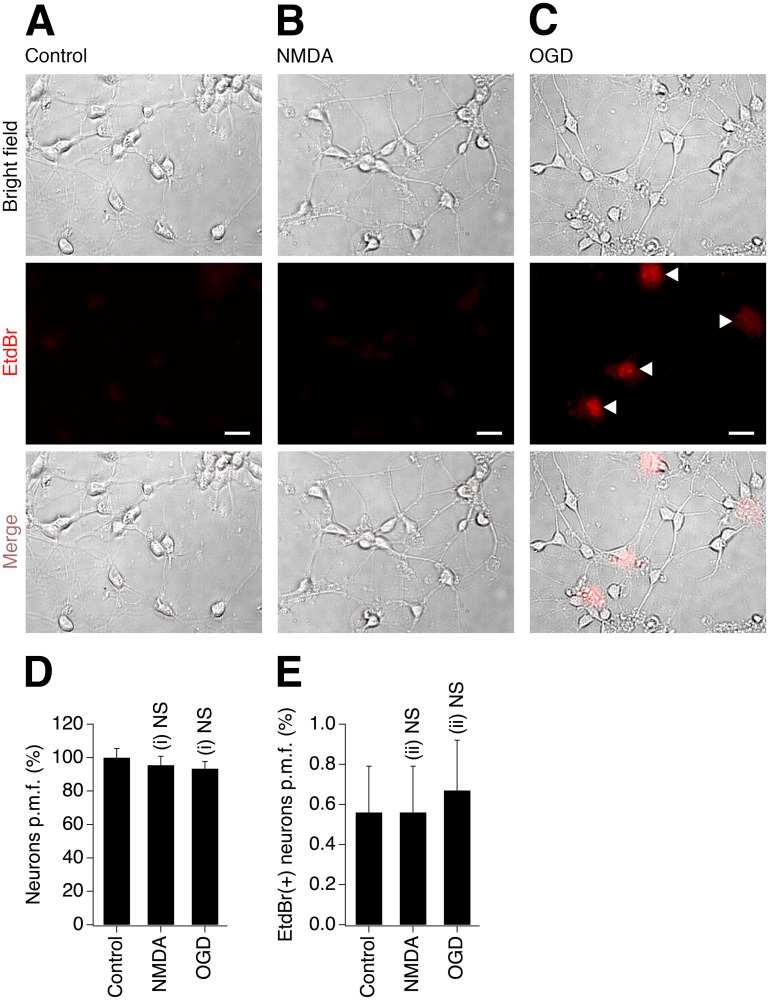
Neither NMDA treatment nor ischemia open neuronal hemichannels. Representative images (**A-C**) and statistical data (**D**, **E**) from EtdBr uptake experiments in WT neuronal cortical cultures are shown. **A-C**, Vast majority of neurons did not show EtdBr staining in control cultures (**A**) and in cultures subjected to NMDAR excitotoxicity (**B**) or OGD (**C**). Some glial cells showed EtdBr uptake following OGD (denoted by arrowheads in the EtdBr image in **C**). **D**, The number of neurons per microscope field (p.m.f.) is presented as percentage of neurons relative to the control. **E**, Percentage of EtdBr-positive neurons of the total number of neurons p.m.f. is shown. Statistical analysis (in **D**, **E**): ANOVA with post hoc Tukey; shown relative to the control; *n* = 30 per group; mean ± SEM. The analysis was done 2 hours post-insult. Calibration bar (**A-C**): 20 μm.

We used neuronal somatosensory cortical cultures prepared from WT mice. Experiments were performed on DIV10, at the time when developing neurons abundantly express endogenous Cx36 and gap junctions [19]. Moreover, the analysis of neuronal EtdBr uptake was done two hours following the insult, when expression of Cx36 is drastically increased following ischemic injury *in vivo* and *in vitro*, prior to the gradual decrease over next several hours [6]. The cultures were subjected to either NMDA (10 μM) or OGD for 30 minutes, followed by washout and the subsequent EtdBr exposure two hours after the insult. On average, 27–29 neurons per microscope field were seen in coverslips under different experimental conditions; only an insignificant (5–7%) reduction in the number of neurons was detected two hours following the insults (Fig 8D). In control cultures, basal level of EtdBr uptake was low and the vast majority of neurons did not show staining (Fig 8A and 8E). Neither NMDA nor OGD insults changed the percentage of neurons demonstrating the EtdBr uptake (Fig 8B,8C and 8E), suggesting the absence of active neuronal hemichannels. Because our primary focus was on examining neuronal EtdBr uptake and the experiments were done in purified neuronal cultures (which contained only small number of glial cells; see Materials and Methods), we did not conduct the analysis of dye uptake by glia. However, we noticed a visible increase in glial EtdBr uptake, particularly following ischemic insult (Fig 8C).

## Discussion

Our previous studies showed that genetic or pharmacological blockade of neuronal (Cx36-containing) gap junctions prevents NMDAR-mediated excitotoxicity [14, 18, 19] and dramatically reduces neuronal death caused by ischemia and TBI [6, 15]. Based on those studies we hypothesized [22, 23] that, following neuronal injury, the major determinant of secondary, glutamate-dependent neuronal death is not overactivation of NMDARs *per se*, but rather the expression of neuronal gap junctions. In the present study, we provide data supporting the critical role of gap junctions in neuronal death. Using WT neuronal cortical cultures, we show that increases and decreases in the level of expression of Cx36-containing gap junctions augment and reduce, respectively, NMDAR-mediated excitotoxicity and ischemic neuronal death.

We also observed that cultures that lack functional gap junctions between neurons (i.e., Cx36 knockout cultures transduced with the control lentivirus or mutant connexin vectors) are not susceptible to NMDAR-mediated excitotoxicity, though they are vulnerable to ischemic neuronal death. The latter presumably is due to direct ischemic injury in neurons (where NMDAR excitotoxicity pays only partial role).

Based upon existing knowledge of the structure and function of gap junctions, there are two competing (though not mutually-exclusive) models by which gap junctions may contribute to cell death: via channel-dependent or channel-independent mechanisms. The first model (the “bystander cell death model”) has been proposed based on experiments in non-neuronal cells and posits that gap junctions contribute to cell death by propagation of a gap junction-permeable death signal(s) from dying cells to healthy cells [28, 35, 36]. According to the second model, connexins are involved in cell death mechanisms independent of their channel activities, interacting directly or indirectly with pro-apoptotic signaling pathways and molecules, as demonstrated for some non-neuronal connexins, including Cx26 and Cx43 (multiple studies, reviewed in [37]). We show here that the contribution of neuronal gap junctions to cell death requires functional, patent channels, but that the connexin isoform making up those channels is not critical. Firstly, the neuronal death phenotype absent in Cx36 knockout neuronal cultures can be rescued by over-expression of Cx36, Cx43 or Cx31. This indicates that, in addition to neuronal Cx36 gap junctions, gap junction channels that are made of connexins typically not present in neurons also promote neuronal loss. Although some of scaffolding components of these different connexins overlap (e.g., zonula occludens-1 for Cx36 and Cx43; [42]), given the heterogeneity of the intracellular domains of these connexins, it is unlikely they all engage the same death-related signal-transduction pathways and/or bind the same scaffolding proteins. This supports the idea for a channel-dependent role for neuronal gap junctions in promotion of neuronal death. Secondly, channel-impermeable gap junctions, created by over-expressing Cx36 or Cx43 with specific amino acid substitutions, were unable to rescue neuronal death in Cx36 knockout cultures. These data support the primacy of channel-dependent mechanism over channel-independent contributions of neuronal gap junctions to neuronal death.

As described above, the simplest mechanistic explanation for the contribution of gap junction channels to cell death is that they allow propagation of pro-death signals between the coupled cells. Some of those death signals have been suggested based on studies with non-neuronal gap junctions. These include inositol-1,4,5-trisphosphate, reactive oxygen and nitrogen species and presumably Ca^2+^ [36, 37]. Such death signals have not been yet identified for neurons. Future studies will address this issue.

It is interesting to note that unitary conductance of Cx36 gap junction channels is about ten times lower than that of Cx43 channels: 10–15 pS and 130 pS, respectively [43, 44] (data for Cx31 do not exist). However, the amounts of neuronal death provided by both channels were comparable (Fig 7A and 7C). The percentage of transduced neurons also was comparable between Cx36- and Cx43-transduced cultures (Fig 1B and 1C). This apparently indicates that the amount of presumptive death signals passing through the pore of Cx36 channel and the diameter of the pore are sufficient to induce death in a given number of neurons. Further increases in the pore’s diameter and/or conductance of death signals (that presumably would occur in Cx43-transduced cultures) do not appear to provide any additional effects on neuronal death.

It should be mentioned, however, that there are several possibilities for the role of gap junctions in neuronal death other than outlined above. The first possibility is that the introduced connexin mutations disrupt some channel-independent, protein-dependent intracellular signaling to death pathways. Given the location of the amino acid substitutions, which were chosen based upon biochemical and structural studies, this explanation is highly unlikely. Employing three different mutations in Cx36 (L10A/Q15A, L10P/Q15P and I22R) and a previously characterized Cx43 mutation (G21R; [31]) with identical result also argues against this possibility.

The second possibility is that the genetic manipulations of Cx36 have an impact on the expression of other genes, which then indirectly influence neuronal death/survival. As discussed above, we show in the present study that up-regulation of Cx36 increases, and down-regulation decreases, neuronal death. Further, over-expression of Cx36 (or even other, non-neuronal connexins) rescues the death phenotype in Cx36-deficient neurons. These findings agree with the previous observations on neuroprotective effects of Cx36 knockout in various models of neuronal injury [6, 14–18]. Importantly, pharmacological blockade of gap junctions has the same neuroprotective effect as genetic does [6, 14]. In addition, developmental up-regulation and down-regulation of Cx36 are associated with, respectively, increased and decreased sensitivity of neurons to NMDAR excitotoxicity [18, 19]. Finally, Cx36 knockout does not cause structural/anatomical alterations and induces only minor changes in the animals’ physiological functions (reviewed in [23]). Given all this, we believe that the effects of genetic manipulations of Cx36 on cell death/survival are induced via change in the amount of electrical synapses between neurons and are not due to any influence on the expression of other genes. Future studies to identify changes in gene expression in neurons following overexpression and knockdown of Cx36 will definitively clarify this issue.

Although Cx36 knockout induces substantial (>95%) loss of neuronal gap junction coupling in the mouse brain [24, 45], there is evidence that a number of other connexins may form gap junctions between neurons [23]. Therefore, the third possibility is that non-Cx36 gap junctions and hemichannels are involved in glutamate- and injury-mediated neuronal death [4, 8]. However, given that genetic elimination of Cx36 prevents NMDAR-mediated excitotoxicity *in vivo* and *in vitro* [14, 18] (and present work), it is difficult to argue a role for other, non-Cx36 gap junctions or hemichannels in NMDAR excitotoxicity. Particularly, in the present study, NMDAR-mediated excitotoxicity was not observed in Cx36-deficient neurons or in neurons expressing mutant Cx36 or Cx43. In addition, in wild-type cultures, neuronal death was prevented by knockdown of Cx36 with shRNA. Further, given the significant level of neuroprotection provided by Cx36 knockout in ischemia, TBI and numerous neuronal injury models *in vitro* [6, 14–16] (also present study), we suggest that, among various neuronal and non-neuronal connexins, Cx36 is the critical player in neuronal death independent of the nature of initiating injury.

The fourth possibility for the contribution of Cx36 to neuronal death is that Cx36 hemichannels, rather than gap junctions, play a role. However, our study revealed low basal EtdBr uptake in control neurons and no increase in uptake two hours after NMDAR-excitotoxicity or ischemia. As stated in the Results section, two hours post-insult is when the increase in expression of neuronal Cx36 peaks following ischemic injury *in vivo* and *in vitro* [6]. Therefore, it would be logical to expect that, if Cx36 hemichannels are activated following insult, their peak activity also coincides with maximal Cx36 protein levels. We did not observe any increase in EtdBr uptake during this period, supporting our previous contention that neuronal hemichannels have little if any role in NMDAR-mediated neuronal death [18]. It is still possible that neuronal hemichannels open at later periods of time, for example two or three days after the insult as it was reported for experiments with continuous treatment of neuronal cultures with pro-inflammatory cytokines [32] or amyloid-β peptide [33]. However, under our experimental conditions, Cx36-dependent neuronal death was observed 24 hours after the insult, suggesting that late opening of Cx36 hemichannels (e.g., even if occurs 2–3 days post-insult) is unlikely to play a role in this neuronal death. Nevertheless, we did see an increase in EtdBr uptake in glial cells two hours following ischemia. This agrees with previous finding by others demonstrating rapid activation of glial connexin hemichannels in experimental conditions related to ischemic injury [46–48].

A final consideration is the role of pannexin channels. Previous studies showed that pannexin channels are expressed in cortical neurons, they open within minutes following insult (including ischemia and activation of NMDARs) and may play a role in injury-mediated neuronal death [48–50]. However, as discussed above, we did not observe any substantial contribution of non-Cx36 factors to neuronal death or an increase in EtdBr uptake, which would have occurred if pannexin channels were active. The approach used in this study was genetic manipulation of Cx36 and our findings do not exclude that, under different experimental conditions (e.g., with manipulation of the expression of pannexins), a role for pannexin channels in neuronal death may be revealed. As for the absence of EtdBr uptake in our study, in relation to the pannexin channel activity this potentially may indicate that the channels are already closed 2 hours post-insult or that other experimental approaches (e.g., release of calcein from loaded neurons [49, 50]) are more suitable to detect the activity of pannexin channels.

In conclusion, this study provides insight into the mechanisms of contribution of neuronal gap junctions in NMDAR-dependent excitotoxicity and ischemic neuronal death. Because clinical trials for NMDAR antagonists as neuroprotective agents largely failed [51], our study suggests that another important therapeutic target for the development of new neuroprotective agents can be neuronal gap junction coupling.
